# Colonic Lipoma Causing Bowel Intussusception: An Up-to-Date Systematic Review

**DOI:** 10.3390/jcm10215149

**Published:** 2021-11-02

**Authors:** Francesco Menegon Tasselli, Fabrizio Urraro, Guido Sciaudone, Giulia Bagaglini, Francesca Pagliuca, Alfonso Reginelli, Franca Ferraraccio, Salvatore Cappabianca, Francesco Selvaggi, Gianluca Pellino

**Affiliations:** 1 Department of Advanced Medical and Surgical Sciences, Università degli Studi della Campania “Luigi Vanvitelli”, 80138 Naples, Italy; francesco.menegontasselli@gmail.com (F.M.T.); guido.sciaudone@unicampania.it (G.S.); giulia.bagaglini@hotmail.it (G.B.); gianluca.pellino@unicampania.it (G.P.); 2 Division of Radiodiagnostic, Università degli Studi della Campania Luigi Vanvitelli, 80138 Naples, Italy; fabrizio.urraro@policliniconapoli.it (F.U.); alfonso.reginelli@unicampania.it (A.R.); salvatore.cappabianca@unicampania.it (S.C.); 3 Division of Pathology, Department of Mental and Physical Health and Rehabilitation Medicine, Vanvitelli University, 80132 Napoli, Italy; frances.pagliuca@gmail.com (F.P.); franca.ferraraccio@unicampania.it (F.F.); 4 Colorectal Surgery, Vall d’Hebron University Hospital, 08035 Barcelona, Spain

**Keywords:** colonic lipoma, colocolic intussusception, surgery, lipoma

## Abstract

Background: Colonic lipomas are rare and can sometimes cause intussusception. The aim of this review was to define the presentation and possible management for colocolic intussusception caused by colonic lipomas. Methods: A systematic search for patients with colocolic intussusception caused by colonic lipoma, including all available reports up to 2021. Epidemiological, clinical, laboratory, and instrumental data and details about the treatments performed were gathered. Results: Colocolic intussusception caused by lipoma is more frequent in women (57%), occurring between 40 and 70 years of age. Up to 83% of patients report abdominal pain, followed by constipation (18%), rectal bleeding (16%), and diarrhea (12%), with abdominal tenderness (37%), and distension in 16%, whereas 24% have a negative exploration. CT (72%) and colonoscopy (62%) are more commonly able to diagnose the entity. The most common location of intussusception is the transverse colon (28%). The surgical operation varies according to the site. The average dimensions of the lipoma are 59.81 × 47.84 × 38.9 mm^3^. Conclusions: A correct preoperative diagnosis of colonic lipoma causing intussusception might not be easy. Despite nonspecific clinical and laboratory presentation, cross-sectional imaging can help differential diagnosis. Surgical treatment depends on the localization.

## 1. Introduction

Lipomas are nonepithelial benign adipose tumors observed throughout the gastrointestinal tract, most frequently located in the colon [1]. Generally, these neoplasms present as a sessile polypoid mass, rarely pedunculated, with dimensions ranging from 2 mm to 30 cm.

In the majority of cases, lipomas are asymptomatic, with the diagnosis being made incidentally during colonoscopy, surgery, or autopsy [2]. However, in some cases, lipomas may present with symptoms, such as abdominal pain, diarrhea, constipation, the mimicking of colon cancer and, occasionally, intussusception [3].

Intestinal intussusception is the invagination of an intestinal loop with a mesenteric fold (intussusceptum) in the lumen of a continuous portion of the intestine (intussuscipiens) following peristalsis [4]. However, in adults, it is rare (1.86 cases/year [5]), and has various clinical presentations compared to the pediatric form (classic triad of abdominal pain, palpable abdominal mass, and hematochezia) [6], making it difficult to diagnose preoperatively, despite the evolution of imaging procedures [7].

Although abdominal computed tomography (CT) has proved useful in diagnosing intussusception in 72% of cases [8,9], it has limited value in discriminating whether the cause is malignant, benign, or idiopathic [10]. Above all, preoperative discrimination between malignant tumors and large lipomas is often impossible, and a diagnosis is only obtained with histological examination [11].

The aim of this study was to define the reported distribution of colonic lipomas, which are the cause of colonic intussusception, in order to identify the best diagnostic and therapeutic procedure for the condition.

## 2. Materials and Methods

### 2.1. Literature Search Strategy

A systematic review was performed in accordance with the Preferred Reporting Items for Systematic Reviews and Meta-Analyses (PRISMA) guidelines. PubMed databases were searched using the date range of January 1900 to June 2021. The key words searched were “colonic” AND “lipoma” AND “intussusception”. One reviewer (FMT) reviewed all the records by title and abstract, followed by a review of the full-text article for those that met the screening criteria. The patient observed by the authors of the current manuscript gave written informed consent for publication.

### 2.2. Eligibility Criteria

Inclusion criteria consisted of colonic intussusception caused by lipoma and treated by endoscopic or surgical intervention. Reports of randomized controlled trials, cohort studies, case–control studies, and case series were included. No language restrictions were applied. Items in which patients were not of adult age were excluded. Items in which the diagnosis of intussusception was not lipoma were excluded.

### 2.3. Data Extraction and Quality Assessment

All articles selected for full-text review were examined by the reviewer (FMT), who decided on inclusion or exclusion and the extraction of the study data. For the purpose of this review, the colonic lipoma was classified by localization along the colon (cecum, ascending colon, transverse colon, descending colon, sigmoid colon, and rectum), by size, and by histological finding. Colonic intussusception was then classified by signs and symptoms, laboratory tests, radiological exams, and the type of intervention (surgical or endoscopic). Information regarding the demographic characteristics of the study subjects, the position of lead points, patient symptoms and physical examinations, laboratory findings, diagnostic methods, operative methods, hospital lengths of stay, and dimensions of the tumor was extracted from each study report and tabulated in Microsoft Excel.

### 2.4. Data Analysis

A single weight-adjusted mean or proportion for each variable or outcome was computed for each item.

## 3. Results

**Figure 1:**
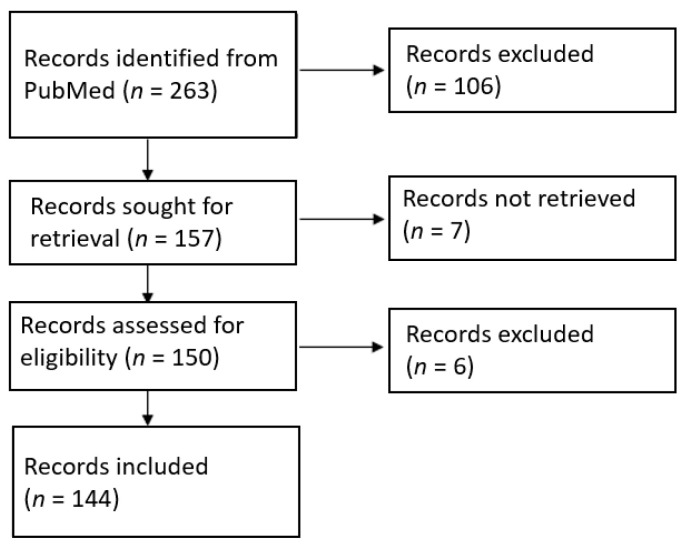
PRISMA flow chart.

A total of 144 full-text articles were included, out of 263 records (Figure 1). All 144 articles were retrospective case series or case reports that included data on 167 eligible patients. There were no randomized controlled or case–control studies. 24 studies were published before 2000, 38 were published between 2000 and 2009, and 82 were published after 2010. The entire list of included articles can be found as Supplementary File S1 and the PRISMA Checklist as Supplementary File S2.

**Figure 2:**
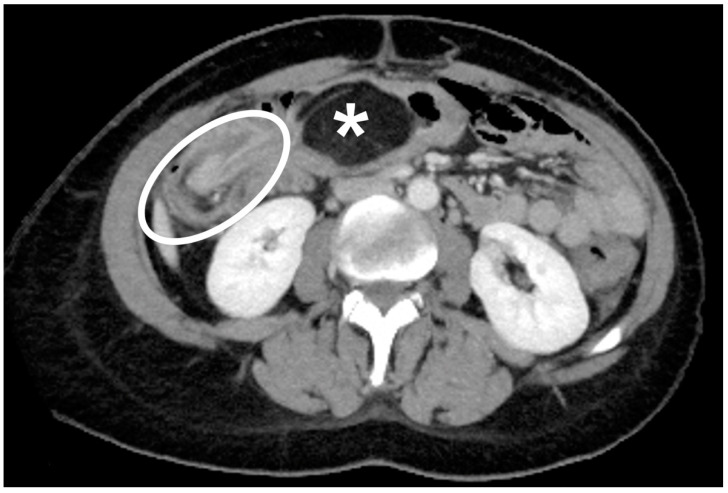
Axial CT scan of the abdomen in portal/venous phase. A rounded, smoothly outlined and sharply demarcated abdominal mass, having fat attenuation mass with fat attenuation (asterisk) is observed. A circumferential thickening in ileocolic intussusception caused by the lipoma can be seen (white circle).

**Figure 3:**
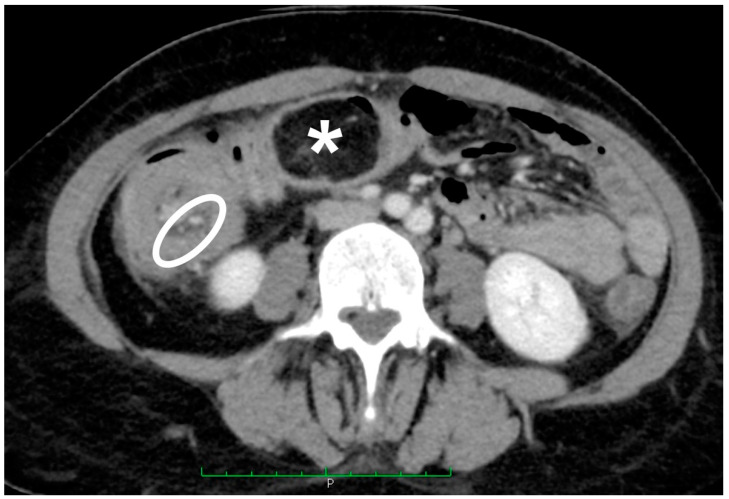
Axial CT scan of the abdomen in portal/venous phase. Circumferential thickening in ileocolic intussusception caused by the lipoma; note the mesenteric fat and vessels (white oval) and the terminal ileum associated with the intussuscipiens (asterisk).

The authors observed a case of colonic lipoma causing intussusception. The data of the patients were pooled in the review. The patient had been reporting diffuse and intermittent abdominal pain for one year. An ultrasound scan was performed, and it showed only biliary sludge. The patient began medical therapy for which she showed improvement. A worsening of the clinical condition in the following two months, associated with the onset of diarrhea, led the patient to be reevaluated for her condition. In this case, a CT scan was performed (Figure 2 and Figure 3).

Given the presence of intussusception, with initial signs of the ischemic suffering of the bowel (Figure 3), immediate surgery was offered.

**Figure 4:**
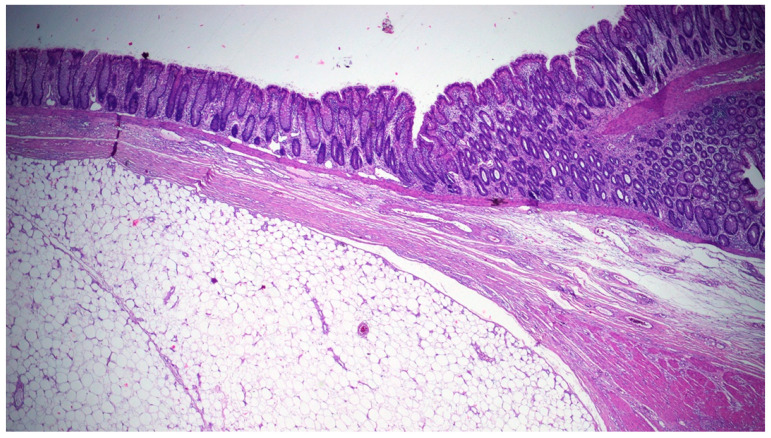
Scanning view showing a well-circumscribed submucosal lesion consisting of mature adipose tissue, surrounded by a rim of muscularis mucosa, and covered by unaltered mucosa.

At surgery, a long colocolic intussusception, with its distal end at the hepatic flexure, was confirmed, and an ileocolic resection and side-to-side anastomosis were performed. The pathology was consistent with a large solitary exophytic mass in the ascending colon, with a uniform yellow cut surface (Figure 4).

**Figure 5:**
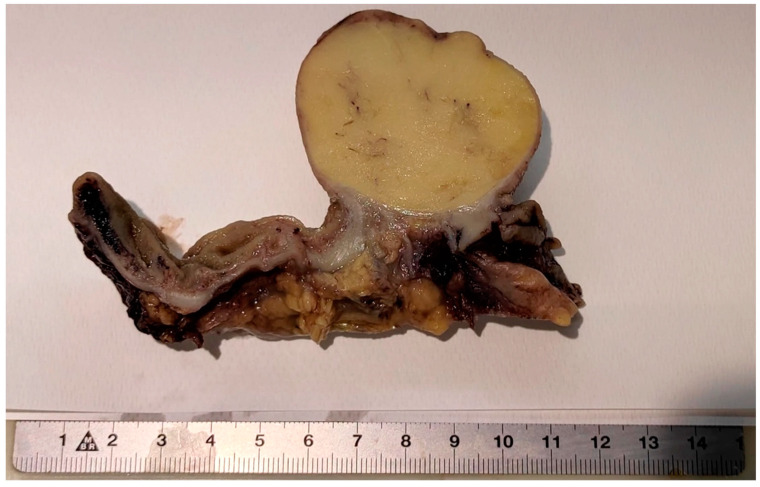
A large well-circumscribed soft mass in the colonic wall. Cut surface is bright yellow and uniform.

The lesion was well-circumscribed, but unencapsulated, involving the submucosa, and measuring 6 cm in its maximum diameter. The overlying mucosa appeared to be intact, with a smooth surface. Upon microscopic examination, a nodular submucosal tumor composed of mature adipocytes, and covered by substantially unaltered mucosa, was seen. The lesion showed extension towards the muscularis propria with pushing borders. Neither cellular atypia nor infiltrative growth were found (Figure 5). These findings were consistent with a lipoma of the submucosa.

### 3.1. Demographic Data

Data were available from 162 patients. The median age of patients was 52 (19–88) years, and 72% of patients presenting with colocolic intussusception from colonic lipoma ranged between 40 and 70 years. Men (70) represented 43% of the cases analyzed and women (92) represented 57%.

### 3.2. Clinical Presentation and Laboratory

Data on reported symptoms and physical explorations were available from 161 and 123 patients, respectively.

The most common symptom reported by patients with colocolic intussusception was abdominal pain (83%), described in 29% of cases as intermittent, and in 17% of cases as crampy. Patients reported that symptoms were related to bowel habits, in particular, constipation (18%), or diarrhea (12%), which in some cases is watery or bloody, or alterations in the bowel movements. Low gastrointestinal bleeding occurred in 16% as rectal bleeding, in 4% as rectal bleeding associated with mucus loss, and in 11% as hematochezia. More nonspecific symptoms, such as nausea, vomiting, weight loss, bloating, and fever occurred in 9, 14, 11, 3, and 2%, respectively. Partial obstruction and obstruction occurred in 4% and 7% of cases, respectively. Patients also reported tenesmus (3%) and dyspepsia (1%). All data are shown in Table 1.

The most frequent clinical sign was tenderness (37%), followed by abdominal distension (16%). Depending on the location of the lipoma, a palpable abdominal mass (16%), a prolapsing mass through the rectum (11%), or a rectal mass (2%) was detected. Voluntary guarding was reported in 4% of cases, while rebound tenderness occurred in 2% of cases. A normal abdomen was found in 24% of cases. The data are shown in Table 2.

Laboratory data were available from 65 cases. Laboratory tests were normal in 61% of cases, WBC were high in 16%, and CRP in 8%. Anemia was present in 14% of the subjects included in the study.

### 3.3. Instrumental Examinations

The instrumental methods used for the identification of colocolic intussusception underlying a colonic lipoma were ultrasound, abdominal X-ray, barium-enema, CT, and colonoscopy. These methods were used in 18, 18, 27, 72, and 62% of cases (based on 156 cases), respectively. 1% of the patients examined did not undergo any preoperative instrumental examination, 28% underwent only one imaging evaluation, 48% underwent two different methods, while the remaining 24% of patients underwent three (19%), four (3%), or five (2%) different tests.

### 3.4. Location and Size of The Lipoma

**Figure 6:**
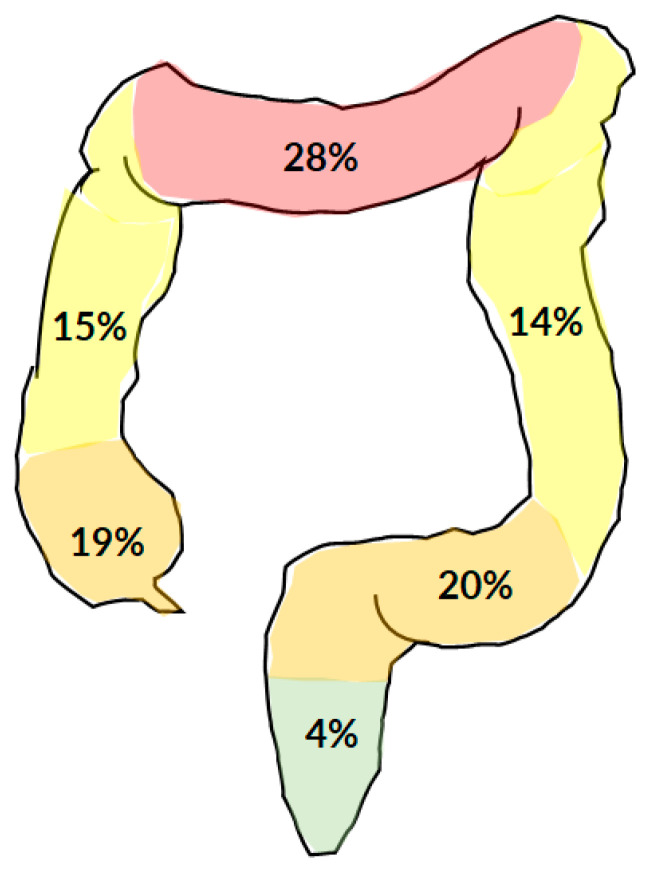
The most typical localization of colonic lipoma causing colocolic intussusception.

The colonic lipoma causing colocolic intussusception was localized more frequently at the transverse colon (28%), followed by the sigmoid (20%), the cecum (19%), the ascending colon (15%), the descending colon (14%), and the rectum (4%) (Figure 6). The average size of a lipoma was 59.81 × 47.84 × 38.90 mm^3^, with a minimum of 15 × 15 × 15 mm^3^, and a maximum of 160 × 110 × 100 mm^3^.

### 3.5. Treatment

Treatment can be performed endoscopically, laparoscopically, or with an open technique. The types of interventions included: endoscopic (10%) or open (8%) polypectomy; right colectomy (32%); extended right colectomy (4%); segmental colectomy (20%); left colectomy (13%); sigmoidectomy (10%); Hartmann sigmoidectomy (1%), and anterior rectal resection (3%). Both open and laparoscopic approaches were used.

The average length of hospital stay was 6.24 (2–18) days.

## 4. Discussion

Colonic lipoma was first described by Bauer in 1757 [12,13]. Since then, various studies have been conducted, classifying lipoma as the third most common benign tumor of the intestine after hyperplastic and adenomatous polyps, with incidence ranging between 0.2 and 4.4% [1,14]. Colonic lipomas are generally small and asymptomatic [13]. Therefore, they are detected incidentally. However, in 25% of cases, there are clinical manifestations [2,13,14], including colocolic intussusception.

Women (57%) are more frequently affected than men (43%), even in the case of symptomatic lipoma [12,15]. Even if intussusception most commonly occurs between 40 and 70 years of age, young patients can also be affected by this problem.

Generally, only 25% of colonic lipoma patients develop symptoms. On the other hand, when a giant lipoma is present, patients who have symptoms reach 75% of cases [16,17].

The main clinical manifestation associated with colocolic intussusception from colonic lipoma appears to be abdominal pain, present in up to 83% of cases. In patients suffering from abdominal pain, 29% of them complain of intermittent pain, while 17% describe a cramp-like pain. Other symptoms often identified by the patients are changes in the bowel habits, in particular: constipation (18%), diarrhea (12%), and, more generally, a change in the bowel movements (8%). There may also be blood (16%) or mucous/blood (4%) losses from the rectum while, on some occasions, gastrointestinal bleeding is limited to less important manifestations, such as hematochezia (11%). The subjects examined also complained of general complaints, such as nausea (9%), vomiting (14%), and weight loss (11%). Depending on the position of the lipoma, the patient may complain of tenesmus (3%), bloating (3%), and up to partial (4%) or complete bowel obstruction (7%).

During physical examination, abdominal tenderness (37%) was the most detected sign, followed by abdominal distension. Surprisingly, 24% of patients, despite an underlying symptomatology, present a completely normal abdomen at physical examination.

In most cases, in patients who came to the hospital for lipoma-induced intussusception, laboratory tests were normal (61%); however, variations in inflammatory indices may be found. Another 14% of patients eventually have anemia.

The clinical presentation of the patient is, therefore, quite vague, and this, together with a nonspecific physical examination and a nonspecific laboratory picture, makes preoperative diagnosis difficult [18,19].

Ultrasound appears to be a rapid and minimally invasive method, which represents the first imaging approach performed by an experienced and skilled operator [20]. The presence of colonic lipoma is indicated by the presence of a well-defined hyperechoic solid mass, with or without minimal vascularization, indicated by the alternation of thin stripes of hypo- and hyperechogenicity [21]. However, elements, such as the small size of the lesion, the patient’s large body mass, and the presence of widespread meteorism, can limit an adequate assessment [22]. Despite the simple execution of the exam, ultrasound is used in a limited percentage of cases, i.e., in 18% of the subjects included in the study. This finding can be explained by the fact that it is difficult to perform the diagnosis of intussusception caused by lipoma, considering that the bowel content can limit the hyperechoic aspects of the lesion, and that the malignant characteristics of a lesion are not always well evident [23], limiting the sensitivity of the method to 33% [5].

Abdominal X-ray is a first-level investigation [24], used in 18% of cases examined. This exam can be negative in the early stages of intussusception, or it can highlight the presence of air-fluid levels, the absence of air in the upper and lower right quadrants, with an increase in soft tissue density, or allow the observation of the “crescent” sign, a typical sign of intussusception, caused by the trapping of gas between the two mucous surfaces of the invaginated bowel [25]. The low sensitivity and specificity explain why this method is little used in the case of suspected intestinal intussusception caused by colonic lipoma [26].

The most used method is the barium-enema study (27%), a method used both for the diagnostic capacity [27], and for the therapeutic effect it can have in cases of intussusception. However, the diagnostic sensitivity is around 36%, not allowing specific diagnoses to be made in the presence of a colon lipoma or any other type of colon cancer.

However, the most used instrumental tests are CT of the abdomen (72%) and colonoscopy (62%).

CT imaging, having a sensitivity of 71–87%, and a specificity of up to 100% [28,29], makes it possible to detect intussusception rather easily as a lesion presenting the typical appearance known as the “target sign” or “sign of donut” [30]. Moreover, if a sufficiently large lesion is present, CT allows for the determination as to whether a lesion is composed of adipose tissue, with values between 40 and 120 Hounsfield units (HU) [31]; if it is associated with an oval shape, suspicion of a lipomatous lesion is strong.

However, particular attention must be paid in making the diagnosis because, in any case, 60–65% of cases of intussusception of the large intestine are underlying malignant lesions [32].

Colonoscopy allows for direct vision of the lesion and biopsies. As in the case of CT, there are pathognomonic signs for lipoma, i.e., the “pillow mark” (soft lesion with a cushion-like mucosal indentation when pressed with closed biopsy forceps), and the “bare fat mark” (leakage of fat after biopsy) [30,33].

Magnetic resonance imaging is very effective in highlighting adipose lesions because of the peculiar characteristics of the signal intensity of this tissue, especially T1-weighted and fat-suppressed images. However, this type of imaging is rarely used to detect and study intestinal neoplastic lesions [34]. In studies included in the current review, this type of examination was used in a limited number of cases.

More than 70% of patients require at least two instrumental investigations before having a diagnosis of intestinal intussusception caused by a lipoma.

The use of different diagnostic techniques depended on the period studied: the barium-enema study, which represented the main instrumental examination performed before 2000, is currently not used (44% pre-2000 vs. 15% post-2000) and has been replaced by computer tomographic examination and the endoscopic exploration of the bowel.

The most conservative option, in cases of certainty of the benign nature of the lesion, involves the simple removal of the lipoma. This procedure can be performed both endoscopically (10% of the cases analyzed) and surgically (8%). In the first case, different techniques are possible, such as the “unroofing” dissection of the lipoma with a loop-assisted snare, the “Ligate and Let Go” technique, the endoscopic mucosal resection (EMR), and the endoscopic submucosal dissection (ESD), all with excellent remission rates [35,36]. In the case of surgery, open (mediated colotomy and colonic enterorrhaphy) or transanal “enucleations” can be performed. However, a more radical surgical resection is recommended when lipomas are complicated by intussusception or intestinal obstruction [31]. The use of minimally invasive techniques allows for an improvement in pain control, reduced hospital stays, and shorter recovery periods [37].

If there is no certain preoperative diagnosis of colon lipoma, or localization of the lipoma and state of the invaginated bowel [29,38], bowel resection is necessary. Surgical resection is recommended by many in the event of intussusception of the colon, especially in elderly patients, because of the high possibility of malignant neoplasm.

The length of hospitalization does not usually exceed one week, depending on the type of approach used and perioperative complications.

Lipomas causing colocolic intussusception are, in most cases (88%), giant lipomas. In almost all cases, the lipomas examined are submucosal and, in minimal part, subserosal, or belonging to the muscularis propria.

## 5. Conclusions

Surgery is currently the most commonly used therapeutic option in intussusception caused by lipoma and, more generally, in adult colonic intussusception. Making a preoperative diagnosis of colonic lipoma causing intussusception is somewhat complex. However, despite the nonspecific clinical-laboratory picture, clinical-instrumental integration can allow a diagnosis to be made, which will still need to be confirmed histologically. In fact, methods have not been developed with sensitivity and specificity such as to guarantee, in all cases, a simple enucleation of the lipoma or minimal resections, without the risk of a malignant diagnosis.

## Figures and Tables

**Table 1:** Summary of symptoms described by the patients.

Symptoms	n.	%
Abdominal pain - Abdominal pain (intermittent) - Abdominal pain (crampy)	134/161 39/134 23/134	83% 29% 17%
Constipation	29/161	18%
Rectal blood loss	26/161	16%
Vomiting	23/161	14%
Diarrhea - Watery diarrhea - Bloody diarrhea	20/161 3/20 2/20	12% 15% 10%
Hematochezia	17/161	11%
Weight loss	18/161	11%
Altered bowel movements	13/161	8%
Nausea	14/161	9%
Obstruction	12/161	7%
Rectal mucus and blood loss	7/161	4%
Partial obstruction	7/161	4%
Tenesmus	5/161	3%
Bloating	5/161	3%
Fever	4/161	2%
Dyspepsia	1/161	1%

**Table 2:** Summary of the most common findings at physical examination.

Signs	n.	%
Tenderness	46/123	37%
Normal exploration	29/123	24%
Distension	20/123	16%
Palpable mass	20/123	16%
Prolapsing mass through the rectum	13/123	11%
Voluntary guarding	5/123	4%
Pain	4/123	3%
Rectal mass	3/123	2%
Rebound tenderness	3/123	2%

## Data Availability

Data are available from the corresponding author.

## Supplementary Materials

The following are available online at https://www.mdpi.com/article/10.3390/jcm10215149/s1. Supplementary File S1: All articles retrieved, Supplementary File S2: PRISMA Checklist.
