# Supplementary material for: Colonic Lipoma Causing Bowel Intussusception: An Up-to-Date Systematic Review
Source: J Clin Med. 2021 Nov 2;10(21):5149. doi: 10.3390/jcm10215149 (PMC8584916; doi:10.3390/jcm10215149)
Supplement: Supplementary file 1 [file jcm-10-05149-s001.zip › All articles retrieved.pdf]

1. Ozen O.; Guler Y.; Yuksel Y. Giant colonic lipoma causing intussusception: CT scan and clinical findings. *Pan Afr Med J*. **2019**, 32, 27. https://doi.org/10.11604/pamj.2019.32.27.18040.

2. Law Y.Y.; Patel R.; Cusick M.; Van Eps J.L. A case of colonic intussusception and obstruction secondary to giant colonic lipoma. *J Surg Case Rep*. **2020**, 2020 (10), rjaa429. https://doi.org/10.1093/jscr/rjaa429.

3. Siamionava Y.; Varabei A.; Makhmudov A. Recurrent colonic intussusception due to a lipoma of the transverse colon at the background of congenital dolichocolon and chronic constipation. *BMJ Case Rep*. **2019**, 12 (8), e230732. https://doi.org/10.1136/bcr-2019-230732.

4. Grasso E.; Guastella T. Giant submucosal lipoma cause colo-colonic intussusception. A case report and review of literature. *Ann Ital Chir*. **2012**, 83 (6), 559-562.

5. Okada M.; Sakamoto H.; Hayashi Y.; Yano T.; Shinozaki S.; Sunada K.; Lefor A.K.; Yamamoto H. Curative endoscopic treatment of intussusception due to a giant colonic lipoma using a wedged balloon and ligation with detachable snares. *Clin J Gastroenterol*. **2019**, 12 (4), 320-324. https://doi.org/10.1007/s12328-019-00943-5.

6. Zenaidi H.; Ismail I.B.; Rekik F.; Aziz M.; Rebii S.; Zoghlami A. Large pedunculated colonic lipoma: a rare cause of colorectal intussusception in adults. *Pan Afr Med J.* **2020**, 36, 200. https://doi.org/10.11604/pamj.2020.36.200.24606.

7. Boyack I.; Vu D.; Patel P.; Opsha O. Colocolic intussusception secondary to submucosal lipoma. *Am J Emerg Med*. **2020**, 38 (8), 1697.e1-1697.e3. https://doi.org/10.1016/j.ajem.2020.04.046.

8. Barry T.M.; Wang C.Q.; Lorch S. Submucosal Lipoma: A Rare Cause of Colonic Intussusception. *Am Surg*. **2020**, 86 (11), 1586-1587. https://doi.org/10.1177/0003134820940287.

9. Chang C.C.; Liu K.L. Colonic lipoma with intussusception. *Mayo Clin Proc*. **2007**, 82 (1), 10. https://doi.org/10.4065/82.1.10.

10. Presti M.E.; Flynn M.F.; Schuval D.M.; Vollmar T.M.; Zotos V.D. Colonic Lipoma With Gastrointestinal Bleeding and Intussusception. *ACG Case Rep J*. **2015**, 2(3), 135-136. https://doi.org/10.14309/crj.2015.32.

11. Shobeirian F.; Mehrnahad M.; Soleimantabar H. Rectal lipoma as a lead point for colo-colonic intussusception. *Radiol Case Rep*. **2018**, 13(2), 431-433. https://doi.org/10.1016/j.radcr.2018.01.012.

12. Siu S.; Oliphant R.; Benstock S.; Keshava A.; Rickard M.J.F.X. Colonic lipoma causing intussusception: a case for colonoscopic surveillance? *ANZ J Surg*. **2019**, 89(4), 428-430. https://doi.org/10.1111/ans.14080.

13. Paškauskas S.; Latkauskas T.; Valeikaitė G.; Paršeliūnas A.; Svagždys S.; Saladžinskas Z.; Tamelis A.; Pavalkis D. Colonic intussusception caused by colonic lipoma: a case report. *Medicina (Kaunas)*. **2010**, 46(7), 477-481.

14. James J.; Strauss P.N. Complicated colonic intussusception. *J Emerg Trauma Shock*. **2012**, 5 (2), 188-189. https://doi.org/10.4103/0974-2700.96493.

15. Mouaqit O.; Hasnai H.; Chbani L.; Oussaden A.; Maazaz K.; Amarti A.; Taleb K.A. Pedunculated lipoma causing colo-colonic intussusception: a rare case report. *BMC Surg*. **2013**, 13, 51. https://doi.org/10.1186/1471-2482-13-51.

16. Kikuchi N.; Miyakura Y.; Takahashi J.; Takayama N.; Tamaki S.; Ishikawa H.; Kakizawa N.; Hasegawa F.; Kikugawa R.; Tsujinaka S.; Lefor A.K.; Rikiyama T. Intussusception secondary to descending colon lipoma presenting with simultaneous acute appendicitis. *J Surg Case Rep*. **2018**, 2018 (7), rjy152. https://doi.org/10.1093/jscr/rjy152.

17. Avilés-Salas A.; Cuéllar-Mendoza M.E. Lipoma submucoso de colon e intususcepción [Submucosal lipoma of the colon with intussusception]. *Acta Gastroenterol Latinoam*. **2012**, 42 (3), 216-219.

18. West C.T.; Pilarski A.; White D.; Ricketts D. An intussuscepting colonic lipoma causing prolapse of the sigmoid colon in an adult. *Br J Hosp Med (Lond)*. **2019**, 80 (3), ii. https://doi.org/10.12968/hmed.2019.80.3.ii.

19. Liang M.M.; Win T.; Rasheed S. A Cecal Lipoma Causing Intussusception; Detected on Routine Outpatient Abdominal Ultrasound. J Med Ultrasound. **2019**, 28 (1), 44-47. https://doi.org/10.4103/JMU.JMU_50_19.

20. Moussally M.; Mokalled I.; Jamali F.; Khalife M.J. Splenic flexure colonic lipoma causing intussusception. JRSM Open. **2021**, 12 (1), 2054270420983088. https://doi.org/10.1177/2054270420983088.

21. Altay C.; Unlu M.; Fuzun M.; Secıl M. Core curriculum illustration: colonic intussusception due to pedunculated lipoma. *Emerg Radiol*. **2020**, 27 (4), 461-462. https://doi.org/10.1007/s10140-018-1613-z.

22. Adachi S.; Hamano R.; Shibata K.; Yoshida S.; Tateishi H.; Kobayashi T.; Hanada M. Colonic lipoma with florid vascular proliferation and nodule-aggregating appearance related to repeated intussusception. *Pathol Int*. **2005**, 55 (3), 160-164. https://doi.org/10.1111/j.1440-1827.2005.01803.x.

23. Bagherzadeh Saba R.; Sadeghi A.; Rad N.; Safari M.T.; Barzegar F. Colonic intussusception in descending colon: An unusual presentation of colon lipoma. *Gastroenterol Hepatol Bed Bench*. **2016**, 9 (Suppl1), S93-S96.

24. Lin C.W.; Hsieh Y.H.; Tzeng J.E.; Tseng K.C. Lipoma-induced colon intussusception. *Endoscopy*. **2009**, 41 (Suppl 2), E14-E15. https://doi.org/10.1055/s-2007-995727.

25. Donovan A.; Abeyasundara S.; Nabi H. Colonoscopic resection of giant colonic lipoma causing subacute large bowel obstruction. *ANZ J Surg*. **2020**, 90 (1-2), E13-E14. https://doi.org/10.1111/ans.15082.

26. Kabaalioğlu A.; Gelen T.; Aktan S.; Kesici A.; Bircan O.; Lüleci E. Acute colonic obstruction caused by intussusception and extrusion of a sigmoid lipoma through the anus after barium enema. *Abdom Imaging*. **1997**, 22 (4), 389-391. https://doi.org/10.1007/s002619900216.

27. Zaghouani Ben Alaya H.; Mallat N.; Manel L.; Majdoub S.; Amara H.; Bakir D.; Kraiem C. Invagination colocolique sur lipome du côlon transverse [Colo-colonic intussusception owing to lipoma of transverse colon]. *Tunis Med*. **2013**, 91 (10), 610-1.

28. Martin P.; Sklow B.; Adler D.G. Large colonic lipoma mimicking colon cancer and causing colonic intussusception. *Dig Dis Sci*. **2008**, 53 (10), 2826-7. https://doi.org/10.1007/s10620-008-0202-5.

29. Espinel J.; Pinedo E.; Rascarachi G. Lipoma gigante de colon e invaginación intestinal [Giant lipoma and intestinal intussusception]. *Rev Esp Enferm Dig*. **2009**, 101 (11), 815-817. https://doi.org/10.4321/s1130-01082009001100013.

30. Basterra Ederra M.; Bolado Concejo F.; Caballero García P.; Oteiza Martínez F. Invaginación intestinal por lipoma de colon de gran tamaño. Tratamiento laparoscópico [Giant lipoma-induced colonic intussusception. Laparoscopic management]. *Gastroenterol Hepatol*. **2011**, 34 (8), 589-590. https://doi.org/10.1016/j.gastrohep.2011.04.009.

31. Twigt B.A.; Nagesser S.K.; Sonneveld D.J. Colo-colonic intussusception caused by a submucosal lipoma: case report and review of the literature. *Case Rep Gastroenterol*. **2007**, 1 (1), 168-173. https://doi.org/10.1159/000112651.

32. Wei R.; Xu W.; Xiao Y.; Zeng F.; Mao S. Laparoscopic segmental resection of the rectum for upper rectal intussusception caused by a giant rectal lipoma: A case report. *Medicine (Baltimore)*. **2018**, 97 (39), e12272. https://doi.org/10.1097/MD.0000000000012272.

33. Toumi O.; Hellara O.; Hammami M.; Mahmoudi A.; Nasr M.; Kallel W.; Zouari K.; Noomen F.; Saffar H.; Hamdi A. Colo-colonic intussusception secondary to a colonic lipoma: report of two cases. *Tunis Med*. **2014**, 92 (11), 700-701.

34. Schopis M.; Yang J. Endoscopic Treatment of Intussusception From Massive Colonic Lipomas via Endoscopic Mucosal Resection: A Case Series. *ACG Case Rep J*. **2019**, 6 (9), e00177. https://doi.org/10.14309/crj.0000000000000177.

35. da Rocha F.F.; Campos M.G. Intussuscepção intestinal por lipoma colocólico [Intestinal intussusception caused by colonic lipoma]. *Rev Assoc Med Bras (1992)*. **2006**, 52 (3), 138. https://doi.org/10.1590/s0104-42302006000300011.

36. Kitamura K.; Kitagawa S.; Mori M.; Haraguchi Y. Endoscopic correction of intussusception and removal of a colonic lipoma. *Gastrointest Endosc*. **1990**, 36 (5), 509-511. https://doi.org/10.1016/s0016-5107(90)71128-5.

37. Lee J.M.; Kim J.H.; Kim M.; Kim J.H.; Lee Y.B.; Lee J.H.; Lim C.W. Endoscopic submucosal dissection of a large colonic lipoma: Report of two cases. *World J Gastroenterol*. **2015**, 21 (10), 3127-3131. https://doi.org/10.3748/wjg.v21.i10.3127.

38. Dolan K.; Khan S.; Goldring J.R. Colo-colonic intussusception due to lipoma. *J R Soc Med*. **1998**, 91 (2), 94. https://doi.org/10.1177/014107689809100217.

39. Miloudi N.; Hefaiedh R.; Khalfallah M.T. Giant lipoma of the transverse colon causing colo-colonic intussusceptions. *J Visc Surg*. **2012**, 149 (6), 421-422. https://doi.org/10.1016/j.jviscsurg.2012.04.006.

40. Shepherd T.; Wazir M.; Cover J. Rare case of adult colocolic intussusception. *BMJ Case Rep*. **2020**, 13 (3), e232761. https://doi.org/10.1136/bcr-2019-232761.

41. Kosaka R.; Noda T.; Tsuboi J.; Tanaka K. Successful endoscopic removal of a large colonic lipoma causing intussusception. *Endoscopy*. **2014**, 46 (Suppl 1 UCTN), E551-E552. https://doi.org/10.1055/s-0034-1377953.

42. Jeong T.G.; Choi S.C.; Seo G.S. [Colocolic intussusception caused by lipoma]. *Korean J Gastroenterol*. **2012**, 59 (5), 386-388. https://doi.org/10.4166/kjg.2012.59.5.386.

43. McKay R. Ileocecal intussusception in an adult: the laparoscopic approach. *JSLS*. **2006**, 10 (2), 250-253.

44. Geraci G.; Pisello F.; Arnone E.; Sciuto A.; Modica G.; Sciumè C. Endoscopic Resection of a Large Colonic Lipoma: Case Report and Review of Literature. *Case Rep Gastroenterol*. **2010**, 4 (1), 6-11. https://doi.org/10.1159/000260053.

45. Mason R.; Bristol J.B.; Petersen V.; Lyburn I.D. Education and imaging. Gastrointestinal: lipoma induced intussusception of the transverse colon. *J Gastroenterol Hepatol*. **2010**, 25 (6), 1177. https://doi.org/10.1111/j.1440-1746.2010.06360.x.

46. Tsai K.J.; Tai Y.S.; Hung C.M.; Su Y.C. Cecal lipoma with subclinical appendicitis: A case report. *World J Clin Cases*. **2019**, 7 (2), 209-214. https://doi.org/10.12998/wjcc.v7.i2.209.

47. Ruiz-Tovar J.; López-Delgado A.; Santos J.E.; Arroyo A.; Calpena R. Tratamiento laparoscópico de invaginación en sigma por lipoma submucoso gigante [Laparoscopic treatment of sigmoid colon intussusception secondary to giant submucosal lipoma]. *Acta Gastroenterol Latinoam*. **2013**, 43 (1), 36-38.

48. Lin I.; Chang W.; Hsu T.; Shih S.; Wang T.E.; Chu C.; Liou T. Lobulated colonic lipoma mimicking carcinoma with intermittent intussusception. *Endoscopy*. **2008**, 40 (Suppl 2), E256-E257. https://doi.org/10.1055/s-2007-966583.

49. Franc-Law J.M.; Bégin L.R.; Vasilevsky C.A.; Gordon P.H. The dramatic presentation of colonic lipomata: report of two cases and review of the literature. *Am Surg*. **2001**, 67 (5), 491-494.

50. Katsinelos P.; Chatzimavroudis G.; Zavos C.; Paroutoglou G.; Papaziogas B.; Kountouras J. A novel technique for the treatment of a symptomatic giant colonic lipoma. *J Laparoendosc Adv Surg Tech A*. **2007**, 17 (4), 467-469. https://doi.org/10.1089/lap.2006.0208.

51. Feo C.V.; Marcello D.; Feo C.F. Laparoscopic treatment of colo-colic intussusception secondary to a lipomatous polyp. *Ann Ital Chir*. **2017**, 6, S2239253X17027189.

52. Guillén-Paredes M.P.; Martínez-Gómez D.; Aguayo-Albasini J.L.; Mengual-Ballester M.; Flores-Pastor B.M. Invaginación colocólica por lipoma [Lipoma causing colo-colonic intussusception]. *Cir Esp*. **2010**, 87 (1), 46-47. https://doi.org/10.1016/j.ciresp.2008.12.017.

53. Hozo I.; Perkovic D.; Grandic L.; Klaudije G.; Simunic M.; Piplovic T. Colonic lipoma intussusception: a case report. *Med Arh*. **2004**, 58 (6), 382-383.

54. Chan K.C.; Lin N.H.; Lien H.C.; Chan S.L.; Yu S.C. Intermittent intussusception caused by colonic lipoma. *J Formos Med Assoc*. **1998**, 97 (1), 63-65.

55. Marra B. Occlusione intestinale da lipoma del colon. A proposito di due casi [Intestinal occlusion due to a colonic lipoma. Apropos 2 cases]. *Minerva Chir*. **1993**, 48 (18), 1035-1039.

56. Lee J.Y.; Ye B.D. [Colonic intussusceptions caused by a giant lipoma]. *Korean J Gastroenterol*. **2012**, 60 (3), 186-189. https://doi.org/10.4166/kjg.2012.60.3.186.

57. Low H.M.; Chinchure D. Clinics in diagnostic imaging (172). Colocolic intussusception with a lipoma as the lead point. *Singapore Med J*. **2016**, 57 (12), 664-668. https://doi.org/10.11622/smedj.2016181.

58. Tony J.; Saji S.; Sandesh K.; Sunilkumar K.; Ramachandran T.M.; Thomas V. External resection of a giant sigmoid lipoma causing colonic intussusception and prolapse through the anal canal. *Trop Gastroenterol*. **2007**, 28 (3), 127-128.

59. Rodríguez-Otero C.; Targarona E.M.; Estalella L.; Martínez C. Invaginación colocólica por lipoma a nivel de anastomosis [Colocolic intussusception due to an anastomotic lipoma]. *Cir Esp*. **2011**, 89 (9), e7. https://doi.org/10.1016/j.ciresp.2010.10.012.

60. Jiménez-Rodríguez R.M.; Serrano-Borrero I.; Díaz-Pavón J.M.; Socas-Macías M.; Vázquez-Monchul J.M. Obstrucción intestinal subaguda debida a intususpección por lipoma colónico [Subacute intestinal obstruction secondary to colonic lipoma intussusception]. *Rev Esp Enferm Dig*. **2008**, 100 (3), 182-183. https://doi.org/10.4321/s1130-01082008000300014.

61. Jelenc F.; Brencic E. Laparoscopically assisted resection of an ascending colon lipoma causing intermittent intussusception. *J Laparoendosc Adv Surg Tech A*. **2005**, 15 (2), 173-175. https://doi.org/10.1089/lap.2005.15.173.

62. Shehzad K.N.; Monib S.; Ahmad O.F.; Riaz A.A. Submucosal lipoma acting as a leading point for colo-colic intussusception in an adult. *J Surg Case Rep*. **2013**, 2013 (10), rjt088. https://doi.org/10.1093/jscr/rjt088.

63. Kuzmich S.; Connelly J.P.; Howlett D.C.; Kuzmich T.; Basit R.; Doctor C. Ileocolocolic intussusception secondary to a submucosal lipoma: an unusual cause of intermittent abdominal pain in a 62-year-old woman. *J Clin Ultrasound*. **2010**, 38 (1), 48-51. https://doi.org/10.1002/jcu.20620.

64. Megaly M.; Yacoub G. Transverse Colon Submucosal Lipoma Presenting With Colocolic Intussusception. *ACG Case Rep J*. **2016**, 3 (3), 158-159. https://doi.org/10.14309/crj.2016.35.

65. Ghidirim G.; Mishin I.; Gutsu E.; Gagauz I.; Danch A.; Russu S. Giant submucosal lipoma of the cecum: report of a case and review of literature. *Rom J Gastroenterol*. **2005**, 14 (4), 393-396.

66. Rogers S.O. Jr.; Lee M.C.; Ashley S.W. Giant colonic lipoma as lead point for intermittent colo-colonic intussusception. *Surgery*. **2002**, 131 (6), 687-688. https://doi.org/10.1067/msy.2002.116409.

67. Atila K.; Terzi C.; Obuz F.; Yilmaz T.; Füzün M. Symptomatic intestinal lipomas requiring surgical interventions secondary to ileal intussusception and colonic obstruction: report of two cases. *Ulus Travma Acil Cerrahi Derg*. **2007**, 13 (3), 227-231.

68. Wild D.; Fiore J.; Guelrud M. Successful endoscopic resection of a giant colonic lipoma causing intussusception. *Gastrointest Endosc*. **2008**, 68 (4), 774-775. https://doi.org/10.1016/j.gie.2008.02.086.

69. Azzopardi C.; Vassallo E.; Grech R.; Mizzi A. Adult colorectal intussusception. *BMJ Case Rep*. **2014**, 2014, bcr2014205597. https://doi.org/10.1136/bcr-2014-205597.

70. Huh K.C.; Lee T.H.; Kim S.M.; Im E.H.; Choi Y.W.; Kim B.K.; Jung D.J.; Choi W.J.; Kang Y.W. Intussuscepted sigmoid colonic lipoma mimicking carcinoma. *Dig Dis Sci*. **2006**, 51 (4), 791-795. https://doi.org/10.1007/s10620-006-3208-x.

71. Fatima H.; Gregory A.; Matthews D. Colocolonic intussusception secondary to an intraluminal lipoma. *Clin Gastroenterol Hepatol*. **2007**, 5 (9), e38. https://doi.org/10.1016/j.cgh.2007.07.002.

72. Chiba T.; Suzuki S.; Sato M.; Tsukahara M.; Saito S.; Inomata M.; Orii S.; Suzuki K. A case of a lipoma in the colon complicated by intussusception. *Eur J Gastroenterol Hepatol*. **2002**, 14 (6), 701-702. https://doi.org/10.1097/00042737-200206000-00018.

73. Alkim C.; Saşmaz N.; Alkim H.; Cağlikülekçi M.; Turhan N. Sonographic findings in intussusception caused by a lipoma in the muscular layer of the colon. *J Clin Ultrasound*. **2001**, 29 (5), 298-301. https://doi.org/10.1002/jcu.1038.

74. Ford K. 4th; Lopez S.; Synghal G.; Fayiga Y.; Carter B.; Kandel A.; Ford K. 3rd. Pedunculated sigmoid lipoma causing colo-colonic intussusception. *Proc (Bayl Univ Med Cent)*. **2021**, 34 (3), 371-372. https://doi.org/10.1080/08998280.2021.1877510.

75. Moussa O.M.; Tee M.; Khan A.U.; Selvasekar C.R. Computerized tomography providing definitive diagnosis of colonic lipoma: a case series. *Surg Laparosc Endosc Percutan Tech*. **2013**, 23 (6), e232-e234. https://doi.org/10.1097/SLE.0b013e31828e3e37.

76. Kaushik R.; Yadav T.D.; Dabra A. A case of sigmoid lipoma presenting with intussusception. *Trop Gastroenterol*. **2001**, 22 (2), 97-98.

77. Zeebregts C.J.; Geraedts A.A.; Blaauwgeers J.L.; Hoitsma H.F. Intussusception of the sigmoid colon because of an intramuscular lipoma. Report of a case. *Dis Colon Rectum*. **1995**, 38 (8), 891-892. https://doi.org/10.1007/BF02049848.

78. Noussias M.; Ward-Mcquaid N. Intussusception Of The Large Bowel In The Adult: Report Of Three Cases With Rare Causes. *Dis Colon Rectum*. **1964**, 7, 189-190. https://doi.org/10.1007/BF02633629.

79. Karaosmanoglu D.; Temizoz O.; Karcaaltincaba M.; Akata D. Sonographic findings of colonic lipoma causing intussusception. *J Ultrasound Med*. **2007**, 26 (11), 1621-1623. https://doi.org/10.7863/jum.2007.26.11.1621.

80. Abu-Dalu J.; Urca I. Lipoma of the colon: report of three cases. *Dis Colon Rectum*. **1972**, 15 (5), 370-372. https://doi.org/10.1007/BF02587414.

81. Rassu P.C.; Bronzino P.; Cassinelli G.; La Spisa C.; Cuneo A.; Partipilo F.; Rusca I.; Boccardo F.; Zoppi S.; Casaccia M. Addome acuto da invaginazione colo-colica: un caso di invaginazione da lipoma del colon [Acute abdomen caused by colo-colic invagination: a case of invagination of a colonic lipoma]. *G Chir*. **2003**, 24 (3), 65-68.

82. Waltermire J.A. Lipoma of the colon with intussusception. *South Med J*. **1977**, 70 (5), 611-612. https://doi.org/10.1097/00007611-197705000-00034.

83. Gürses B.; Kabakci N.; Akyuz U.; Pata C.; Taviloglu K.; Kovanlikaya I. Imaging features of a cecal lipoma as a lead point for colo-colonic intussusception. *Emerg Radiol*. **2008**, 15 (2), 133-136. https://doi.org/10.1007/s10140-007-0641-x.

84. Tzilinis A.; Fessenden J.M.; Ressler K.M.; Clarke L.E. Transanal resection of a colonic lipoma; mimicking rectal prolapse. *Curr Surg*. **2003**; 60 (3), 313-314. https://doi.org/10.1016/S0149-7944(02)00729-8.

85. Crozier F.; Portier F.; Wilshire P.; Navarro-Biou A.; Panuel M. Diagnostic par scanner d'une invagination colocolique sur lipome du côlon gauche [CT scan diagnosis of colo-colic intussusception due to a lipoma of the left colon]. *Ann Chir*. **2002**, 127 (1), 59-61. https://doi.org/10.1016/s0003-3944(01)00670-8.

86. Vekić B.; Živić R. Pedunculated obstructive lipoma of the ileocecal valve: a case report. *Srp Arh Celok Lek*. **2014**, 142 (11-12), 721-723. https://doi.org/10.2298/sarh1412721v.

87. Welch C.E.; Wyman S.M.; et al. Submucous lipoma of transverse colon; with previous intussusception. *N Engl J Med*. **1948**, 239 (23), 897. https://doi.org/10.1056/NEJM194812022392308.

88. Rutherford C.L.; Alkhaffaf B.; Massa E.; Turner P. Colo-colic intussusception secondary to lipomatous polyp in an adult. *BMJ Case Rep*. **2013**, 2013, bcr2012008037. https://doi.org/10.1136/bcr-2012-008037.

89. Ladurner R.; Mussack T.; Hohenbleicher F.; Folwaczny C.; Siebeck M.; Hallfeld K. Laparoscopic-assisted resection of giant sigmoid lipoma under colonoscopic guidance. *Surg Endosc*. **2003**, 17 (1), 160. https://doi.org/10.1007/s00464-002-4232-3.

90. Taylor B.A.; Wolff B.G. Colonic lipomas. Report of two unusual cases and review of the Mayo Clinic experience; 1976-1985. *Dis Colon Rectum*. **1987**, 30 (11), 888-893. https://doi.org/10.1007/BF02555431.

91. Yang Y.W.; Liang J.T. Colocolonic intussusception with a leading point. *Clin Gastroenterol Hepatol*. **2011**, 9 (4), e29. https://doi.org/10.1016/j.cgh.2010.10.027.

92. Dultz L.A.; Ullery B.W.; Sun H.H.; Huston T.L.; Eachempati S.R.; Barie P.S.; Shou J. Ileocecal valve lipoma with refractory hemorrhage. *JSLS*. **2009**, 13 (1), 80-83.

93. Kansoun A.; Mohtar I.A.; Bahmad M.; Houcheimi F.; Maanieh N.; Hazim M.; Rahal K.; Amiry A.R.; Hoteit A.; Saeed A.A.; Fakhruddin N.; Wehbe M. Colo-colic intussusception secondary to colon lipoma: A case report. *Int J Surg Case Rep*. **2021**, 81, 105695. https://doi.org/10.1016/j.ijscr.2021.105695.

94. Wang T.K. Adult descending colocolic intussusception caused by a large lipoma. *Gastroenterol Jpn*. **1992**, 27 (3), 411-413. https://doi.org/10.1007/BF02777762.

95. Kastanakis M.; Anyfantakis D.; Symvoulakis E.K.; Katsougris N.; Papadomichelakis A.; Kokkinos I.; Petrakis G.; Bobolakis E. Cecal lipoma presenting as acute intestinal obstruction in an elderly woman: a case report. *Case Rep Surg*. **2013**, 2013, 926514. https://doi.org/10.1155/2013/926514.

96. Siddiqui M.N.; Garnham J.R. Submucosal lipoma of the colon with intussusception. *Postgrad Med J*. **1993**, 69 (812), 497. https://doi.org/10.1136/pgmj.69.812.497.

97. Barchetti F.; Al Ansari N.; De Marco V.; Caravani F.; Broglia L. Giant lipoma of descending colon diagnosed at CT: report of a case. *Eur Rev Med Pharmacol Sci*. **2010**, 14 (6), 573-575.

98. Mandal S.; Kawatra V.; Dhingra K.K.; Gupta P.; Khurana N. Lipomatous Polyp Presenting With Intestinal Intussusception in Adults: Report of Four Cases. *Gastroenterology Res*. **2010**, 3 (5), 229-231. https://doi.org/10.4021/gr232e.

99. Croome K.P.; Colquhoun P.H. Intussusception in adults. *Can J Surg*. **2007**, 50 (6), E13-E14.

100. Wilhelmsen M.; Mynster T. Prolapse of submucous lipoma of the sigmoid colon. *Pol Przegl Chir*. **2012**, 84 (2), 102-104. https://doi.org/10.2478/v10035-012-0017-0.

101. Pack G.T.; Booher R.J. Intussuscepting submucous lipoma of right colon. *Surg Clin North Am*. **1947**, 27, 361-372. https://doi.org/10.1016/s0039-6109(16)32092-8.

102. Voron T.; Soyer P.; Dohan A. A rare cause of acute bowel obstruction in adult. *Clin Res Hepatol Gastroenterol*. **2014**, 38 (3), 241-242. https://doi.org/10.1016/j.clinre.2013.10.007.

103. Hodgman C.G.; Lantz E.J.; Maus T.P.; Conley C.R. Computed tomography of intussusception due to colon lipoma. *J Comput Assist Tomogr*. **1987**, 11 (4), 740-741. https://doi.org/10.1097/00004728-198707000-00042.

104. Kumar L.; Laksman T.K. Giant submucosal lipomatous polyp causing perforation of sigmoid colon: a case report and review of literature. *J Clin Diagn Res*. **2015**, 9 (1), PD22-PD24. https://doi.org/10.7860/JCDR/2015/10554.5468.

105. Walters J.B.; Sundaram V.; Yadav D. Electronic clinical challenges and images in GI. *Gastroenterology*. **2009**, 136 (2), e5-e6. https://doi.org/10.1053/j.gastro.2008.09.006.

106. Eustace S.; Murray J.G.; O'Connell D. Sonographic diagnosis of colonic lipoma-induced intussusception. *J Clin Ultrasound*. **1993**, 21 (7), 472-474. https://doi.org/10.1002/jcu.1870210713.

107. Sohn J.; Knox R.D.; Gilmore A. Laparoscopic Resection of an Acute Rectosigmoid Intussusception Due to a Giant Pedunculated Lipoma. *Cureus*. **2021**, 13 (3), e13798. https://doi.org/10.7759/cureus.13798.

108. Gordon R.S.; O'Dell K.B.; Namon A.J.; Becker L.B. Intussusception in the adult--a rare disease. *J Emerg Med*. **1991**, 9 (5), 337-342. https://doi.org/10.1016/0736-4679(91)90377-r.

109. Ferraris R.; Fornaro R.; Torelli I.; Parodi G.; Rollandi G.A. A proposito di un caso di lipoma sottomucoso del cieco [A case of submucous lipoma of the cecum]. *Minerva Chir*. **1987**, 42 (1-2), 97-100.

110. Chen R.; Zhao H.; Sang X.; Mao Y.; Lu X.; Yang Y. Severe adult ileosigmoid intussusception prolapsing from the rectum: A case report. *Cases J*. **2008**, 1 (1), 198. https://doi.org/10.1186/1757-1626-1-198.

111. Dallos A. Submucous lipoma of transverse colon with intussusception. *Am J Dig Dis*. **1947**, 14 (11), 345-348. https://doi.org/10.1007/BF03001109.

112. Liessi G.; Pavanello M.; Cesari S.; Dell'Antonio C.; Avventi P. Large lipomas of the colon: CT and MR findings in three symptomatic cases. *Abdom Imaging*. **1996**, 21 (2), 150-152. https://doi.org/10.1007/s002619900032.

113. Parmar J.H.; Lawrence R.; Ridley N.T. Submucous lipoma of the ileocaecal valve presenting as caecal volvulus. *Int J Clin Pract*. **2004**, 58 (4), 424-425. https://doi.org/10.1111/j.1368-5031.2004.00063.x.

114. Alponat A.; Kok K.Y.; Goh P.M.; Ngoi S.S. Intermittent subacute intestinal obstruction due to a giant lipoma of the colon: a case report. *Am Surg*. **1996**, 62 (11), 918-921.

115. Mussler A.; Schröder R.J. Durch eine intestinale intraluminale Raumforderung ausgelöste abdominale Schmerzen [Abdominal pain caused by an intestinal intraluminal space occupying lesion]. *Radiologe*. **2008**, 48 (12), 1161-1163. https://doi.org/10.1007/s00117-008-1701-2.

116. Brünner H.; Ehlert C.P.; Loth R. Submuköse Lipome des Dickdarms [Submucous lipoma of the colon]. *Dtsch Med Wochenschr*. **1973**, 98 (21), 1064-1066. https://doi.org/10.1055/s-0028-1106966.

117. Furste W.; Hadder T. Submucosal lipoma of the ileocecal valve causing intussusception and obstruction. *Dis Colon Rectum*. **1958**, 1 (4), 262-265. https://doi.org/10.1007/BF02617058.

118. Tsiaousidou A.; Chatzitheoklitos E.; Hatzis I.; Alatsakis M.; Katsourakis A. Giant transmural lipoma of the sigmoid colon. *Hippokratia*. **2012**, 16 (3), 278-279.

119. Zhang X.; Ouyang J.; Kim Y.D. Large ulcerated cecal lipoma mimicking malignancy. *World J Gastrointest Oncol*. **2010**, 2 (7), 304-306. https://doi.org/10.4251/wjgo.v2.i7.304.

120. Gys B.; Haenen F.; Gys T. Ileocolic Intussusception Caused by a Giant Ulcerating Lipoma of Bauhin's Valve: an Unusual Cause of Intestinal Obstruction in the Adult. *Indian J Surg*. **2015**, 77 (Suppl 1), 1-2. https://doi.org/10.1007/s12262-013-1023-0.

121. Cordeiro J.; Cordeiro L.; Pôssa P.; Candido P.; Oliveira A. Intestinal intussusception related to colonic pedunculated lipoma: A case report and review of the literature. *Int J Surg Case Rep*. **2019**, 55, 206-209. https://doi.org/10.1016/j.ijscr.2019.01.042.

122. Meunier M.; Limgba A.; Schumacker I.; Mehdi A. Intussusception related to transverse colonic lipoma: a case report and review of the literature. *Acta Chir Belg*. **2020**, 1-5. https://doi.org/10.1080/00015458.2020.1739843.

123. M'rabet S.; Jarrar M.S.; Akkari I.; Abdelkader A.B.; Sriha B.; Hamila F.; Letaief R.; Jazia E.B. Colonic intussusception caused by a sigmoidal lipoma: A case report. *Int J Surg Case Rep*. **2018**, 50, 1-4. https://doi.org/10.1016/j.ijscr.2018.06.009.

124. Pintor-Tortolero J.; Martínez-Núñez S.; Tallón-Aguilar L.; Padillo-Ruiz F.J. Colonic intussusception caused by giant lipoma: a rare cause of bowel obstruction. *Int J Colorectal Dis*. **2020**, 35 (10), 1973-1977. https://doi.org/10.1007/s00384-020-03629-4.

125. de Figueiredo L.O.; Garcia D.P.C.; Alberti L.R.; Paiva R.A.; Petroianu A.; Paolucci L.B.; Costa M.R.L.G. Colo-colonic intussusception due to large submucous lipoma: A case report. *Int J Surg Case Rep*. **2016**, 28, 107-110. https://doi.org/10.1016/j.ijscr.2016.09.006.

126. Ávila F.; Pereira J.R.; Duarte M.A. Colonic Intussusception Caused by Colonic Lipoma. *GE Port J Gastroenterol*. **2016**, 23 (5), 264-266. https://doi.org/10.1016/j.jpge.2016.04.004.

127. Mohamed M.; Elghawy K.; Scholten D.; Wilson K.; McCann M. Adult sigmoidorectal intussusception related to colonic lipoma: A rare case report with an atypical presentation. *Int J Surg Case Rep*. **2015**, 10, 134-137. https://doi.org/10.1016/j.ijscr.2015.03.035.

128. Zhou X.C.; Hu K.Q.; Jiang Y. A 4-cm lipoma of the transverse colon causing colonic intussusception: A case report and literature review. *Oncol Lett*. **2014**, 8 (3), 1090-1092. https://doi.org/10.3892/ol.2014.2278.

129. Allos Z.; Zhubandykova D. Large benign submucosal lipoma presented with descending colonic intussusception in an adult. *Am J Case Rep*. **2013**, 14, 245-249. https://doi.org/10.12659/AJCR.883975.

130. Panahi S.E.; Mehanna D. Adult intussusception involving colonic lipoma: a case study. *ANZ J Surg*. **2019**, 89 (6), E272-E273. https://doi.org/10.1111/ans.14340.

131. Kwag S.J.; Choi S.K.; Jung E.J.; Jung C.Y.; Jung S.H.; Park T.J.; Ju Y.T. Surgical strategy for colonic intussusception caused by a giant colonic lipoma: a report of two cases and a review of the literature. *Ann Coloproctol*. **2014**, 30 (3), 147-50. https://doi.org/10.3393/ac.2014.30.3.147.

132. Atmatzidis S.; Chatzimavroudis G.; Patsas A.; Papaziogas B.; Kapoulas S.; Kalaitzis S.; Ananiadis A.; Makris J.; Atmatzidis K. Pedunculated cecal lipoma causing colo-colonic intussusception: a rare case report. *Case Rep Surg*. **2012**, 2012, 279213. https://doi.org/10.1155/2012/279213.

133. Casiraghi T.; Masetto A.; Beltramo M.; Girlando M.; Di Bella C. Intestinal Obstruction Caused by Ileocolic and Colocolic Intussusception in an Adult Patient with Cecal Lipoma. *Case Rep Surg*. **2016**, 2016, 3519606. https://doi.org/10.1155/2016/3519606.

134. Yamamoto K.; Ikeya T.; Shiratori Y. Endoscopic unroofing and mucosal resection for a large colonic lipoma with intussusception: an effective hybrid technique. *VideoGIE*. **2020**, 6 (4), 190-192. https://doi.org/10.1016/j.vgie.2020.11.018.

135. Son D.N.; Jung H.G.; Ha D.Y. Laparoscopic surgery for an intussusception caused by a lipoma in the ascending colon. *Ann Coloproctol*. **2013**, 29 (2), 80-82. https://doi.org/10.3393/ac.2013.29.2.80.

136. Yoldas T.; Karaca A.C.; Ozturk S.; Unver M.; Calıskan C.; Korkut M.A. A rare cause of colonic obstruction "colonic intussusception": report of two cases. *Case Rep Surg*. **2015**, 2015, 465374. https://doi.org/10.1155/2015/465374.

137. Costeira F.S.; Gonçalves M.; Esteves C.; Rebelo A.; Leite C. Colonic Lipoma as a Cause of Colocolic Intussusception. *GE Port J Gastroenterol*. **2020**, 28 (1), 73-75. https://doi.org/10.1159/000508294.

138. Boyce S.; Khor Y.P. A colonic submucosal lipoma presenting with recurrent intestinal obstruction attacks. *BMJ Case Rep*. **2009**, 2009, bcr11.2008.1199. https://doi.org/10.1136/bcr.11.2008.1199.

139. Howard N.; Pranesh N.; Carter P. Colo-colonic intusussception secondary to a lipoma. *Int J Surg Case Rep*. **2012**, 3 (2), 52-54. https://doi.org/10.1016/j.ijscr.2011.10.011.

140. Lee C.S.; Lee M.J.; Kim K.L.; Kim Y.S.; Baik G.H.; Kim J.B.; Kim D.J.; Han S.H. A case of giant lipoma causing chronic recurrent intussusception of the colon. *Clin Endosc*. **2012**, 45 (2), 165-168. https://doi.org/10.5946/ce.2012.45.2.165.

141. El Tinay O.Y.; Khan I.A.; Noureldin O.H.; Al Boukai A.A. Caecal lipoma causing colo-colonic intussusception. *Saudi J Gastroenterol*. **2003**, 9 (3), 145-147.

142. Chehade H.H.; Zbibo R.H.; Nasreddine W.; Abtar H.K. Large ileocecal submucosal lipoma presenting as hematochezia; a case report and review of literature. *Int J Surg Case Rep*. **2015**, 10, 1-4. https://doi.org/10.1016/j.ijscr.2015.03.007.

143. Ongom P.A.; Wabinga H.; Lukande R.L. A 'giant' intraluminal lipoma presenting with intussusception in an adult: a case report. *J Med Case Rep*. **2012**, 6, 370. https://doi.org/10.1186/1752-1947-6-370.

144. Bromberg S.H.; Zampieri J.C.; Cavalcanti L.A.; Waisberg J.; Barreto E.; de Godoy A.C. Lipomas colorretais: correlação anatomoclínica de 29 casos [Colorectal lipomas: anatomoclinical study of 29 cases]. *Rev Assoc Med Bras (1992)*. **1997**, 43 (4), 319-325. https://doi.org/10.1590/s0104-42301997000400008.
